# Supplementary material for: Umbrella Review of Systematic Reviews and Meta-Analyses on the Consumption of Different Food Groups and the Risk of Overweight and Obesity
Source: Nutrients. 2025 Feb 13;17(4):662. doi: 10.3390/nu17040662 (PMC11857968; doi:10.3390/nu17040662)

nutrients MDPI

Supplementary data

Table of content

Table S1: Serving sizes used in the dose-response estimates. 2

Search string........ 2

Conversion equations 5

Table S2: Overview of the most comprehensive and up-to-date meta-analyses on associations between food groups and incidence of overweight and obesity 6

Table S3: Overview of all meta-analyses on associations between food groups and incidence of overweight/obesity for high versus low comparison 7

Table S4: Overview of all meta-analyses on associations between food groups and incidence of overweight and obesity per serving 9

Table S5: Overview of all meta-analyses on associations between food groups and incidence of overweight and obesity for non-linear dose-response 11

Table S6: Overview of the quality assessment of the systematic reviews and meta-analysis following the AMSTAR-2 [33] 12

Figure S1: Associations between food groups (high versus low) and incidence of obesity in the most comprehensive and up-to-date meta-analyses. 13

Figure S2: Associations between food groups (per serving) and incidence of obesity in the most comprehensive and up-to-date meta-analysis. 14

Table S1: Serving sizes used in the dose-response estimates.

Food group	Serving size
Whole grains	30 grams
Vegetables	100 grams
Fruits	80 grams
Legumes	50 grams
Nuts	28 grams
Refined grains	30 grams
Sugar-sweetened beverages	250 ml
Total dairy (in milk equivalents)	200 grams

Search string

Ovid MEDLINE(R) Epub Ahead of Print and In-Process, In-Data-Review & Other Non-Indexed Citations and Daily <June 11, 2024>

Date: 12.06.2024

1 food/ or bread/ or exp dairy products/ or exp dietary carbohydrates/ or exp dietary fats/ or exp dietary proteins/ or exp eggs/ or flour/ or food, processed/ or fruit/ or exp meat/ or molasses/ or nuts/ or seeds/ or exp edible grain/ or exp vegetables/ 630555

2 (food group* or bread* or grain* or cereal* or dairy product* or milk or cheese or yogurt or yoghurt or dietary carbohydrate* or dietary fat* or oil or oils or dietary protein* or egg or eggs or flour or processed food* or fruit or fruits or berry or berries or citrus or meat* or fish or sea food* or chicken or molasses or sugar or sugars or sugar-sweetened beverage* or nut or nuts or seed or seeds or vegetable* or legume* or peas or soy or pulses or bean* or lentil*).ti,ab,kf. 1498144

3 1 or 2 1787305

4 Obesity/ or Overweight/ or Overnutrition/ 241090

5 (obese or obesity or obesitas or overweight or overnutrition or body weight disorder*).ti,ab,kf. 428267

6 4 or 5 468562

7 eating/ or drinking/ or food preferences/ 86222

8 (intake* or consum* or eat* or diet*).ti,ab,kf. 1483796

9 7 or 8 1509620

10 3 and 6 and 9 37171

11 meta-analysis/ or "systematic review"/ 350886

12 ((systematic* adj3 (review* or overview*)) or (integrative adj3 (review* or overview*)) or (collaborative adj3 (review* or overview*)) or (meta analy* or metanaly* or meta-analy* or metaanaly* or systematic review*)).ti,ab,kf. 506890

13 11 or 12 536200

14 10 and 13 1181

https://ovidsp.ovid.com/ovidweb.cgi?T=JS&NEWS=N&PAGE=main&SHAREDSEARCHID=5BDjKRCl2AU2HmKgWtkAZoQAtBNVW47pITqlECp9uUEjlYFdHbUkrw9vKZQm1N43X

Embase <1974 to 2024 June 11>

Date: 12.06.2024

1 food/ or exp bakery product/ or exp bran/ or exp dairy product/ or exp edible oil/ or exp egg/ or fat/ or exp flour/ or exp food grain/ or exp fruit/ or margarine/ or exp meat/ or exp noodle/ or exp nut/ or pasta/ or exp poultry product/ or exp processed food/ or exp sea food/ or exp soy food/ or exp vegetable/ or exp whole food/ 895484

2 (food group* or bread* or grain* or cereal* or dairy product* or milk or cheese or yogurt or yoghurt or dietary carbohydrate* or dietary fat* or oil or oils or dietary protein* or egg or eggs or flour or processed food* or fruit or fruits or berry or berries or citrus or meat* or fish or sea food* or chicken or molasses or sugar or sugars or sugar-sweetened beverage* or nut or nuts or seed or seeds or vegetable* or legume* or peas or soy or pulses or bean* or lentil*).ti,ab,kf. 1654015

3 1 or 2 2057278

4 food intake/ or drinking/ or eating/ or fish consumption/ or fruit consumption/ or meat consumption/ or vegetable consumption/ 231117

5 (intake* or consum* or eat* or diet*).ti,ab,kf. 1877862

6 4 or 5 1933324

7 exp obesity/ or overnutrition/ or body weight disorder/ 709955

8 (obese or obesity or obesitas or overweight or overnutrition or body weight disorder*).ti,ab,kf. 638054

9 7 or 8 847211

10 3 and 6 and 9 55354

11 exp meta analysis/ or "systematic review"/ 606361

12 ((systematic* adj3 (review* or overview*)) or (integrative adj3 (review* or overview*)) or (collaborative adj3 (review* or overview*)) or (meta analy* or metanaly* or meta-analy* or metaanaly* or systematic review*)).ti,ab,kf. 631251

13 11 or 12 775700

14 10 and 13 2242

https://ovidsp.ovid.com/ovidweb.cgi?T=JS&NEWS=N&PAGE=main&SHAREDSEARCHID=3UIxc6Dy5ow674j9mmH9TxYMqtIFSjn551mNYLR4zHIyz28wwBeFSgHQgWtghYPWb

Epistemonikos (Epistemonikos foundation**)**

Date: 12.06.2024

Title/abstract searches:

("food group" or "foods groups" or bread* or grain* or cereal* or "dairy product" or "dairy products" or milk or cheese or yogurt or yoghurt or "dietary carbohydrate" or "dietary carbohydrates" or "dietary fat" or "dietary fats" or oil or oils or "dietary protein" or "dietary proteins" or egg or eggs or flour or "processed food" or "processed foods" or fruit or fruits or berry or berries or citrus or meat* or fish or "sea food" or "sea foods" or chicken or molasses or sugar or sugars or "sugar-sweetened beverage" or "sugar-sweetened beverages" or nut or nuts or seed or seeds or vegetable* or legume* or peas or soy or pulses or bean* or lentil*)

AND
(intake* or consum* or eat* or diet*)
AND
(obese or obesity or obesitas or overweight or overnutrition or "body weight disorder" or "body weight disorders")

Broad synthesis: 62
https://tinyurl.com/36csxs73
Structured summary: 1
https://tinyurl.com/yeysz27n
Systematic reviews: 637
https://tinyurl.com/3kj3zyn5
Total: 700

Web of Science Core Collection
Date: 12.06.2024

Entitlements:
- WOS.SCI: 1945 to 2024
- WOS.AHCI: 1975 to 2024
- WOS.ESCI: 2019 to 2024
- WOS.SSCI: 1956 to 2024

Searches:
#1
Search: TS=((("food group*" or bread* or grain* or cereal* or "dairy product*" or milk or cheese or yogurt or yoghurt or "dietary carbohydrate*" or "dietary fat*" or oil or oils) NEAR/2 (intake* or consum* or eat* or diet*)))
Results: 81590
#2
Search: TS=((("dietary protein*" or egg or eggs or flour or "processed food*" or fruit or fruits or berry or berries or citrus or meat* or fish or "sea food*" or chicken or molasses) NEAR/2 (intake* or consum* or eat* or diet*)))
Results: 110301
#3
Search: TS=(((sugar or sugars or "sugar-sweetened beverage*" or nut or nuts or seed or seeds or vegetable* or legume* or peas or soy or pulses or bean* or lentil*) NEAR/2 (intake* or consum* or eat* or diet*)))
Results: 55182
#4
Search: #1 OR #2 OR #3 Results: 215269

#5
Search: TS=((obese or obesity or obesitas or overweight or overnutrition or "body weight disorder*"))
Results: 574795

#6
Search: TI=(((systematic* NEAR/2 (review* or overview*)) or (integrative NEAR/2 (review* or overview*)) or (collaborative NEAR/2 (review* or overview*)) or ("meta analy*" or metanaly* or meta-analy* or metaanaly* or "systematic review*"))) Results: 441470

#7
Search: #4 AND #5 AND #6 Results: 677
https://www.webofscience.com/wos/woscc/summary/33989ebf-be36-4426-a3c6-8eeeb4a8fcfd-ea529e45/relevance/1

Comments:
Web of Science does not use subject headings for indexing. Therefore, we used proximity operators and specific search fields for free text word searches, for reducing the number of irrelevant hits.
Line 1-3: free text word searches for food groups and intake are combined with proximity operator. This means words from both elements must be close to each other with up to 2 words between. Line 6: Study design was searched in only title. Search in Topic would result in many other document types.

Conversion equations

The reported OR was converted to RR using the following equation:

$$RR = \frac{OR}{(1 - Pref) + (Pref * OR)}$$

In this equation Pref = Prevalence of the outcome in the reference group (the non-exposed prevalence). The non-exposed prevalence number was set to 57% [32].

The converted relative risk ratios were calculated with the following equation:

$$RR\ standardized = RR\ reported^{\frac{\text{standardized dose}}{reported\ dose}}$$

Table S2: Overview of the most comprehensive and up-to-date meta-analyses on associations between food groups and incidence of overweight and obesity

Author (Year)	Outcome	Food group	Comparison	Studies/Participants/Events	AMSTAR2	Results	Age (range)	Country/region
Schlesinger (2018)	Overweight /obesity	Dairy	HL	6 / 206,756 / 65,857	High	0.96 [0.88, 1.06] ($I^2 = 0.82$)	18-100	Spain, Denmark, US, South Korea, Germany
Schlesinger (2018)	Overweight /obesity	Dairy	PS (serving size = 200g)	6 / 206,756 / 65,857	High	0.97 [0.93, 1.01] ($I^2 = 0.79$)	18-100	Spain, Denmark, US, South Korea, Germany
Schlesinger (2018)	Overweight /obesity	Fruit	HL	4 / 152,777 / 25,828	High	0.88 [0.80, 0.96] ($I^2 = 0.76$)	18-84	US, Sweden, Germany, Spain
Schlesinger (2018)	Overweight /obesity	Fruit	PS (serving size = 80g)	4 / 152,777 / 25,828	High	0.94 [0.89, 1.00] ($I^2 = 0.89$)	18-84	US, Sweden, Germany, Spain
Schlesinger (2018)	Overweight /obesity	Legumes	HL	1 / 35,515 / 9253	High	0.87 [0.81, 0.94] (I^2 = NA)	24+	US, Germany
Schlesinger (2018)	Overweight /obesity	Legumes	PS (serving size = 50g)	1 / 35,515 / 9253	High	0.88 [0.84, 0.93] (I^2 = NA)	24+	US, Germany
Nishi (2019)	Overweight /obesity	Nuts	HL	5 / 520,331 / 91,256	High	0.93 [0.88, 0.98] ($I^2 = 0.90$)	37-55	Europe, US
Nishi (2019)	Overweight /obesity	Nuts	PS (serving size = 28g)	5 / 520,331 / 91,256	High	0.95 [0.94, 0.96] ($I^2 = 0.89$)	37-55	Europe, US
Rouhani (2013)	Overweight /obesity	Processed meat	HL	5 / 127,079 / NA	Low	1.18 [0.88, 1.41] ($I^2 = 0.68$)		
Schlesinger (2018)	Overweight /obesity	Red meat	HL	1 / 19,885 / 7183	High	1.23 [1.07, 1.41] (I^2 = NA)	18-80	Spain, US
Schlesinger (2018)	Overweight /obesity	Refined grains	HL	3 / 107,642 / 10,596	High	1.11 [0.85, 1.45] ($I^2 = 0.84$)	38-84	Spain, US

Schlesinger (2018)	Overweight /obesity	Refined grains	PS (serving size = 30g)	3 / 107,642 / 10,596	High	1.05 [1.00, 1.10] (I^2 = 0.61)	38-84	Spain, US
Santos (2021)	Overweight /obesity	SSBs	HL	2 / 64,379 / NA	High	1.17 [1.03, 1.33] (I^2 = 0.30)	21-69	US, Korea, Central and Eastern Europe
Santos (2021)	Overweight /obesity	SSBs	PS (serving size = 250g)	4 / 64,379 / NA	High	1.17 [1.10, 1.25] (I^2 = 0.36)	21-69	US, Korea, Central and Eastern Europe
Schlesinger (2018)	Overweight /obesity	Vegetables	HL	3 / 162,262 / 39,114	High	0.93 [0.83, 1.03] (I^2 = 0.66)	19-74	US, Japan, Germany, Spain
Schlesinger (2018)	Overweight /obesity	Vegetables	PS (serving size = 100g)	3 / 162,262 / 39,114	High	0.98 [0.93, 1.03] (I^2 = NA)	19-74	US, Japan, Germany, Spain
Schlesinger (2018)	Overweight /obesity	Whole grains	HL	5 / 185,527 / 18,087	High	0.85 [0.79, 0.91] (I^2 = 0.00)	21-84	Spain, US, Australia
Schlesinger (2018)	Overweight /obesity	Whole grains	PS (serving size = 30g)	5 / 185,527 / 18,087	High	0.93 [0.89, 0.96] (I^2 = 0.00)	21-84	Spain, US, Australia

HL=high versus low; PS=per serving; US=United States

Table S3: Overview of all meta-analyses on associations between food groups and incidence of overweight/obesity for high versus low comparison

Author (Year)	Food group	Type of study	Studies/Participants/Events	AMSTAR-2	Results	Heterogeneity	Age (range)	Country/region
Schlesinger (2018)	Dairy	Cohort	6 / 206756 / 65857	High	0.96 [0.88, 1.06]	0.82	18-100	Spain, Denmark, US, South Korea, Germany
Schlesinger (2018)	Fruit	Cohort	4 / 152777 / 25828	High	0.88 [0.8, 0.96]	0.76	18-84	US, Sweden, Germany Spain

Schlesinger (2018)	Legumes	Cohort	1 / 35515 / 9253	High	0.87 [0.81, 0.94]	NA	24+	US, Germany
Nishi (2019)	Nuts	Cohort	5 / 520331 / 91256	High	0.93 [0.88, 0.98]	0.9	37-55	Europe, US
Schlesinger (2018)	Nuts	Cohort	3 / 460602 / 42991	High	0.91 [0.8, 1.03]	0.25	20-70	Spain, US, Europe, Germany
Rouhani (2013)	Processed meat	Cohort	5 / 127079 / NA	Low	1.18 [0.88, 1.41]	0.68		
Rouhani (2013)	Red meat	Cohort	11 / 127079 / NA	Low	1.14 [0.98, 1.28]	0		
Schlesinger (2018)	Red meat	Cohort	1 / 19885 / 7183	High	1.23 [1.07, 1.41]	NA	18-80	Spain, US
Schlesinger (2018)	Refined grains	Cohort	3 / 107642 / 10596	High	1.11 [0.85, 1.45]	0.84	38-84	Spain, US
Qin (2019)	SSBs	Cohort	7 / 56579 / 11821	High	1.2 [1.1, 1.31]	0.01	18-84	US, Spain, Denmark, Finland, Japan, France, UK, Thailand, Mexico, Korea, Singapore, China
Ruanpeng (2015)	SSBs	RCT & cohort	6 / 39536 / NA	Moderate	1.2 [1.08, 1.32]	0.57	18-84	Spain, US
Santos (2021)	SSBs	Cohort	2 / 64379 / NA	High	1.17 [1.03, 1.33]	0.3	21-69	US, Korea, Central and Eastern Europe
Schlesinger (2018)	SSBs	Cohort	3 / 59521 / 18436	High	1.2 [1.01, 1.43]	0.23	18-80	US, Spain, South Korea, Norway
Schlesinger (2018)	Vegetables	Cohort	3 / 162262 / 39114	High	0.93 [0.83, 1.03]	0.66	19-74	US, Japan, Germany, Spain
Schlesinger (2018)	Whole grains	Cohort	5 / 185527 / 18087	High	0.85 [0.79, 0.91]	0	21-84	Spain, US, Australia

SSB=Sugar-sweetened beverages; NA=Not Available; US=United States

Table S4: Overview of all meta-analyses on associations between food groups and incidence of overweight and obesity per serving

Author (Year)	Food group	Type of study	Studies/Participants/ Events	AMSTAR -2	Results (original)	Results (converted)	Heterogeneity	Age (range)	Country
Feng (2021)	Dairy	Cohort	5 / 31054 / 11103	High	0.75 [0.6, 0.92]	0.75 [0.6, 0.92]	0.93	NA	Korea, US and Spain
Schlesinger (2018)	Dairy	Cohort	6 / 206756 / 65857	High	0.97 [0.93, 1.01]	0.97 [0.93, 1.01]	0.79	18-100	Spain, Denmark, US, South Korea, Germany
Schlesinger (2018)	Fruit	Cohort	4 / 152777 / 25828	High	0.93 [0.86, 1]	0.94 [0.89, 1]	0.89	18-84	US, Sweden, Germany Spain
Schlesinger (2018)	Legumes	Cohort	1 / 35515 / 9253	High	0.88 [0.84, 0.93]	0.88 [0.84, 0.93]	NA	24+	US, Germany
Nishi (2019)	Nuts	Cohort	2 / 1297 / 536	High	0.72 [0.65, 0.8]	0.72 [0.65, 0.8]	0.62	37-55	Europe, US
Li (2017)	Nuts	RCT & cohort	3 / 254779 / 34602	High	0.95 [0.89, 1.02]	0.95 [0.9, 1.02]	0.74	24-67	Canada, US, Spain, Australia, New Zealand, Turkey, South Africa, China, Taiwan, Iran, Brazil, India and Pakistan
Li (2017)	Nuts	RCT & cohort	3 / 254779 / 34602	High	0.97 [0.95, 0.98]	0.97 [0.95, 0.98]	0		
Nishi (2019)	Nuts	Cohort	5 / 520331 / 91256	High	0.95 [0.94, 0.96]	0.95 [0.94, 0.96]	0.89	37-55	Europe, US
Schlesinger (2018)	Nuts	Cohort	3 / 460602 / 42991	High	0.78 [0.58, 1.06]	0.78 [0.58, 1.06]	0.64	20-70	Spain, US, Europe and Germany

Schlesinger (2018)	Refined grains	Cohort	3 / 107642 / 10596	High	1.05 [1, 1.1]	1.05 [1, 1.1]	0.61	38-84	Spain, US
Qin (2019)	SSBs	Cohort	7 / 56579 / 11821	High	1.12 [1.05, 1.19]	1.12 [1.05, 1.19]	0.68	18-84	US, Spain, Denmark, Finland, Japan, France, UK, Thailand, Mexico, Korea, Singapore, China
Santos (2021)	SSBs	Cohort	4 / 64379 / NA	High	1.17 [1.1, 1.25]	1.17 [1.1, 1.25]	0.36	21-69	US, Korea, Central and Eastern Europe
Schlesinger (2018)	SSBs	Cohort	3 / 59521 / 18436	High	1.05 [1, 1.11]	1.05 [1, 1.11]	0.33	18-80	US, Spain, South Korea, Norway
Schlesinger (2018)	Vegetables	Cohort	3 / 162262 / 39114	High	0.98 [0.93, 1.03]	0.98 [0.93, 1.03]	NA	19-74	US, Japan, Germany, Spain
Schlesinger (2018)	Whole grains	Cohort	5 / 185527 / 18087	High	0.93 [0.89, 0.96]	0.93 [0.89, 0.96]	0	21-84	Spain, US, Australia

RCT=Randomized controlled trial; NA=Not available; US=United States

Table S5: Overview of all meta-analyses on associations between food groups and incidence of overweight and obesity for non-linear dose-response

Author (Year)	Food group	Studies/Participants/Events	Type of study	AMSTAR2	Range
Feng (2021)	Dairy	5/31054/11103	Cohort	High	[0 - 600]
Schlesinger (2018)	Dairy	6/206756/65857	Cohort	High	[0 - 700]
Schlesinger (2018)	Fruit	4/152777/25828	Cohort	High	[0 - 280]
Nishi (2019)	Nuts	5/520331/91256	Cohort	High	[0 - 14]
Schlesinger (2018)	Nuts	3/460602/42991	Cohort	High	[0 - 30]
Schlesinger (2018)	Refined grains	3/107642/10596	Cohort	High	[0 - 170]
Qin (2019)	SSBs	7/56579/11821	Cohort	High	[0 - 900]
Schlesinger (2018)	SSBs	3/59521/18436	Cohort	High	[0 - 1000]
Schlesinger (2018)	Vegetables	3/162262/39114	Cohort	High	[0 - 500]
Schlesinger (2018)	Whole grains	5/185527/18087	Cohort	High	[0 - 200]

SSB=Sugar-sweetened beverages

Table S6: Overview of the quality assessment of the systematic reviews and meta-analysis following the AMSTAR-2 [33]

Author (year)	1	2	3	4	5	6	7	8	9	10	11	12	13	14	15	16	AMSTAR-2
Schlesinger (2018)	Yes	Yes	Yes	Yes	Partly	Yes	Yes	Yes	Yes	No	Yes	Yes	Yes	Yes	NR	Yes	High
Eslami (2019)	Yes	Partly	Yes	Yes	No	No	Yes	Yes	Yes	No	No	NR	Yes	NR	NR	Yes	Critical low
Feng (2022)	Yes	Yes	Yes	Yes	Yes	Yes	Partly	Yes	Yes	No	Yes	NR	Yes	Yes	Yes	Yes	High
Li (2018)	Yes	Partly	Yes	Yes	Yes	Yes	Partly	Yes	Yes	No	Yes	Yes	Yes	Yes	Yes	Yes	High
Nishi (2021)	Yes	Partly	Yes	Yes	Yes	Yes	Yes	Yes	Yes	Yes	Yes	NR	Yes	Yes	Yes	Yes	High
Qin (2020)	Yes	Partly	Yes	Yes	Yes	Yes	Partly	Yes	Yes	No	Yes	NR	Yes	Yes	Yes	Yes	High
Rouhani (2014)	Yes	No	Yes	Yes	No	No	Partly	Yes	No	No	Yes	NR	Yes	Yes	Yes	Yes	Critical low
Ruanpeng (2017)	Yes	Partly	Yes	Yes	Yes	Yes	Partly	Yes	Yes	No	Yes	Yes	Yes	Yes	Yes	Yes	Moderate
Santos (2022)	Yes	Yes	Yes	Yes	Yes	Yes	Yes	Yes	Yes	No	Yes	NR	Yes	Yes	Yes	Yes	High
Louie (2011)	Yes	No	Yes	Yes	Yes	Yes	No	Yes	No	No	NR	NR	No	NR	NR	Yes	Critical low
Vasanti (2022)	No	No	Yes	No	No	No	No	Yes	Yes	No	NR	NR	Yes	NR	NR	Yes	Critical low
Nour (2018)	Yes	Partly	Yes	Yes	Yes	Yes	Yes	Yes	Yes	No	NR	NR	No	NR	Yes	Yes	Low
Trumbo (2014)	Yes	No	Yes	No	No	No	No	Yes	No	No	NR	NR	Yes	NR	NR	Yes	Critical low

NR=Not relevant. 1: Did the research questions and inclusion criteria for the review include the components of PICO? 2: Did the report of the review contain an explicit statement that the review methods were established prior to the conduct of the review and did the report justify any significant deviations from the protocol? 3: Did the review authors explain their selection of the study designs for inclusion in the review? 4: Did the review authors use a comprehensive literature search strategy? 5: Did the review authors perform study selection in duplicate? 6: Did the review authors perform data extraction in duplicate? 7: Did the review authors provide a list of excluded studies and justify the exclusions? 8: Did the review authors describe the included studies in adequate detail? 9: Did the review authors use a satisfactory technique for assessing the risk of bias (RoB) in individual studies that were included in the review? 10: Did the review authors report on the sources of funding for the studies included in the review? 11: If meta-analysis was performed, did the review authors use appropriate methods for statistical combination of results? 12: If meta-analysis was performed, did the review authors assess the potential impact of RoB in individual studies on the results of the meta-analysis or other evidence synthesis? 13: Did the review authors account for RoB in primary studies when interpreting/discussing the results of the review? 14: Did the review authors provide a satisfactory explanation for, and discussion of, any heterogeneity observed in the results of the review? 15: If they performed quantitative synthesis did the review authors carry out an adequate investigation of publication bias (small study bias) and discuss its likely impact on the results of the review? 16: Did the review authors report any potential sources of conflict of interest, including any funding they received for conducting the review

Figure S1: Associations between food groups (high versus low) and incidence of obesity in the most comprehensive and up-to-date meta-analyses.

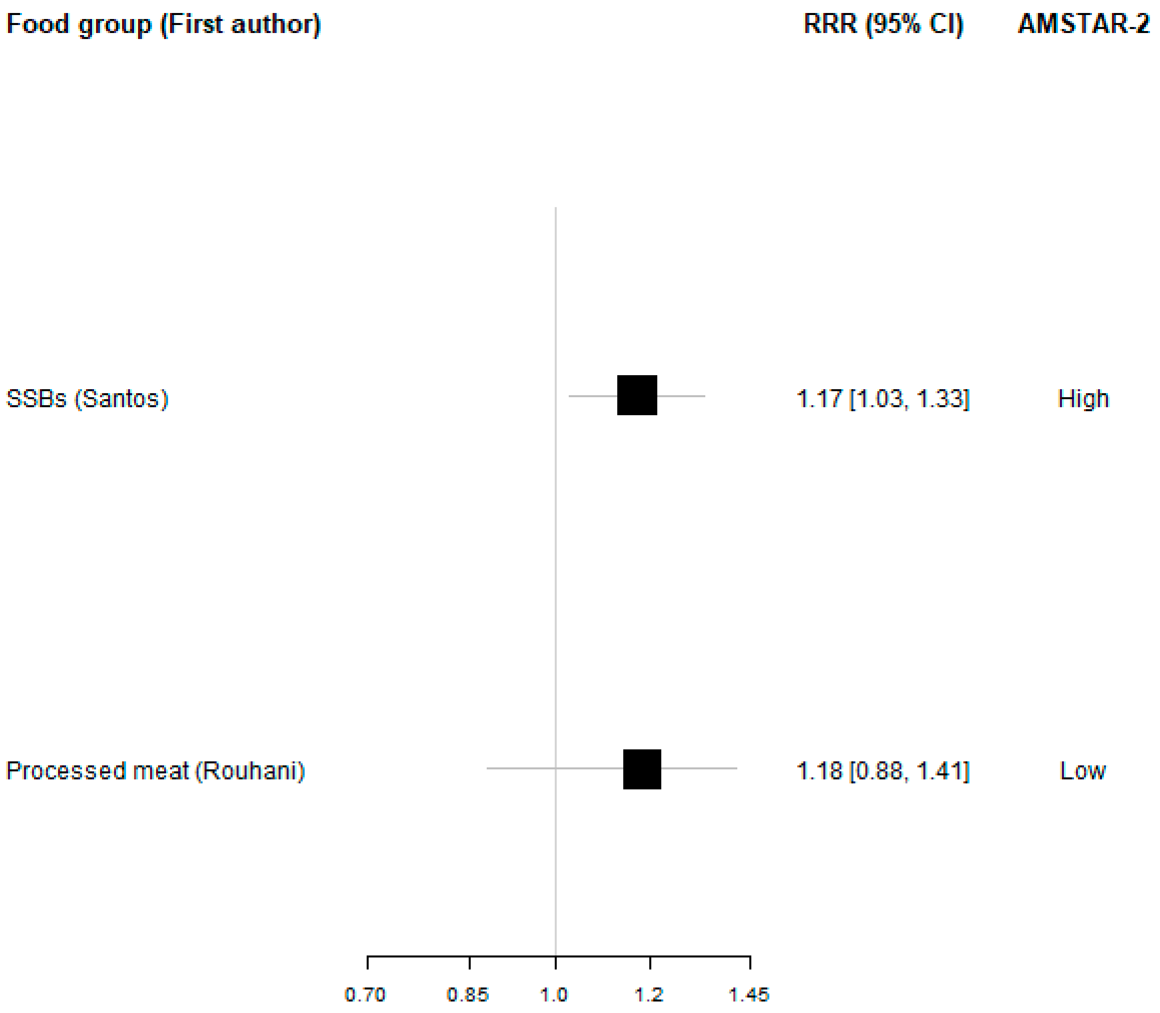

Figure S2: Associations between food groups (per serving) and incidence of obesity in the most comprehensive and up-to-date meta-analysis.

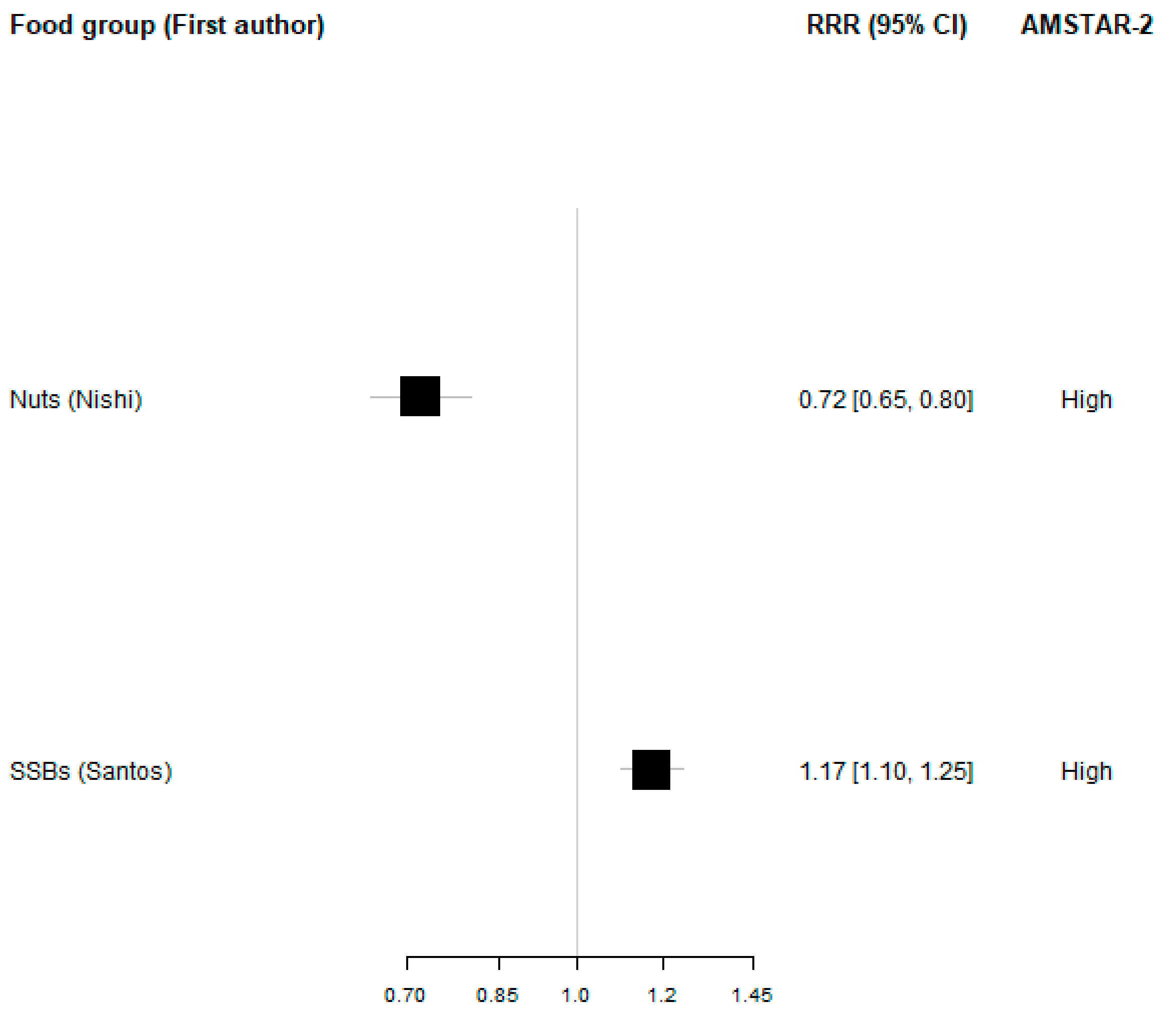

Supplement: Supplementary file 1 [file nutrients-17-00662-s001.zip › nutrients-3450665-supplementary.pdf]
